# ﻿*Mezcala* – a new segregate genus of mimosoid legume (Leguminosae, Caesalpinioideae, mimosoid clade) narrowly endemic to the Balsas Depression in Mexico

**DOI:** 10.3897/phytokeys.205.78297

**Published:** 2022-08-22

**Authors:** Colin E. Hughes, Jens J. Ringelberg, Melissa Luckow, José Luis Contreras Jiménez

**Affiliations:** 1 Department of Systematic & Evolutionary Botany, University of Zurich, Zollikerstrasse 107, 8008 Zurich, Switzerland University of Zürich Zurich Switzerland; 2 School of Integrative Plant Science, Plant Biology Section, Cornell University, 215 Garden Avenue, Roberts Hall 260, Ithaca, NY 14853, USA Cornell University Ithaca United States of America; 3 Facultad de Arquitectura, Benemérita Universidad Autónoma de Puebla, 4 Sur 104, Col. Centro, CP 72000, Puebla, Mexico Benemérita Universidad Autónoma de Puebla Puebla Mexico

**Keywords:** *Desmanthus*, Fabaceae, generic delimitation, *Kanaloa*, monophyly, taxonomy

## Abstract

Recent results have demonstrated that the genus *Desmanthus* is non-monophyletic because the genus *Kanaloa* is nested within it, with a single species, *Desmanthusbalsensis* placed as sister to the clade comprising *Kanaloa* plus the remaining species of *Desmanthus*. Here we transfer *D.balsensis* to a new segregate genus *Mezcala*, discuss the morphological features supporting this new genus, present a key to distinguish *Mezcala* from closely related genera in the Leucaena subclade, and provide a distribution map of *M.balsensis*.

## ﻿Introduction

In the 35 years since *Desmanthusbalsensis* J.L. Contr. was first described (Contreras 1986) and the more than 20 years since the monograph of the genus *Desmanthus* Willd. was published (Luckow 1993), discovery and description of the monospecific Hawaiian endemic genus *Kanaloa* Lorence and K.R. Wood (Lorence and Wood 1994) and assembly of molecular phylogenetic evidence (Luckow et al. 2003; Hughes et al. 2003; Luckow et al. 2005; Ringelberg et al. 2022), have demonstrated that the genus *Desmanthus* is non-monophyletic. To remedy this non-monophyly, we here segregate *D.balsensis* as a new genus, *Mezcala*, thereby rendering *Desmanthus* s.s. monophyletic.

**Figure 1.**
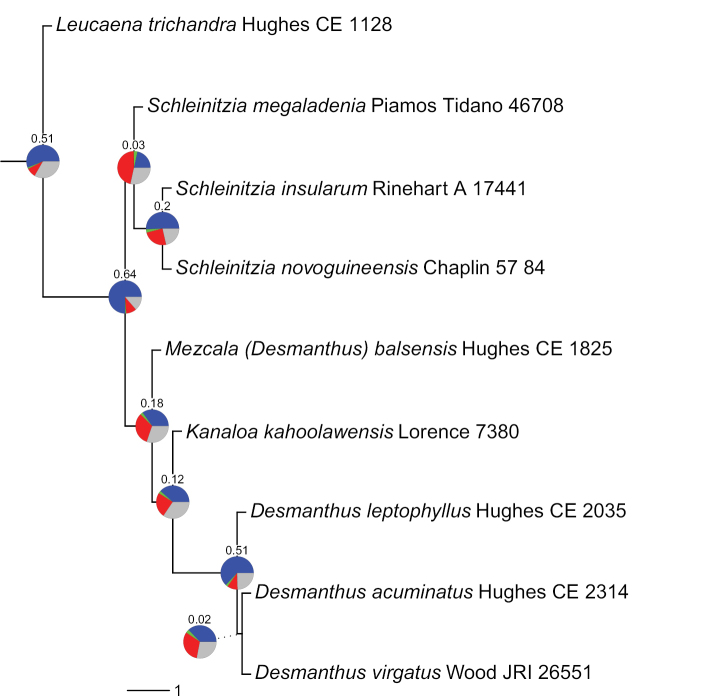
Phylogeny showing sister group relationships of the genera of the informal Leucaena group based on analysis of 997 nuclear gene sequences. The root of the phylogeny is indicated with an arbitrary branch length. Pie charts show the fraction of gene trees supporting that bipartition in blue, the fraction of gene trees supporting the most likely alternative configuration in green, the fraction of gene trees supporting additional conflicting configurations in red, and the fraction of uninformative gene trees in grey. Numbers above pie charts are Extended Quadripartition Internode Certainty scores. Branch lengths are expressed in coalescent units, and terminal branches were assigned an arbitrary uniform length for visual clarity, see Ringelberg et al. (2022).

The molecular evidence for the non-monophyly of *Desmanthus* with *Kanaloa* nested within the genus and *D.balsensis* as sister to the clade comprising *Kanaloa* and the rest of *Desmanthus*, presented by Ringelberg et al. (2022), is compelling as it is based on DNA sequences of 997 nuclear genes obtained via targeted enrichment (hybrid capture, or Hybseq) using a slightly modified version of the *Mimobaits* bait set of Koenen et al. (2020). This large phylogenomic dataset yields a phylogeny that receives maximal bootstrap and posterior probability support in concatenated phylogenetic analyses and shows a high fraction of gene trees supporting this species tree topology (Fig. 1; Ringelberg et al. 2022). Furthermore, analysis of accompanying plastome DNA sequence data obtained from off-target DNA sequence reads from the Hybseq data, confirms this non-monophyly of *Desmanthus* (Ringelberg et al. 2022). This non-monophyly was already hinted at in previous phylogenetic analyses based on small numbers of traditional DNA sequence loci (ITS, *trnL-trnF*, *trnK-matK*), which showed that *D.balsensis* is an outlier in the genus, but that the relationships between *D.balsensis*, *Kanaloa* and the rest of *Desmanthus* were either weakly supported or formed a polytomy (Hughes et al. 2003; Luckow et al. 2003; Luckow et al. 2005).

**Figure 2.**
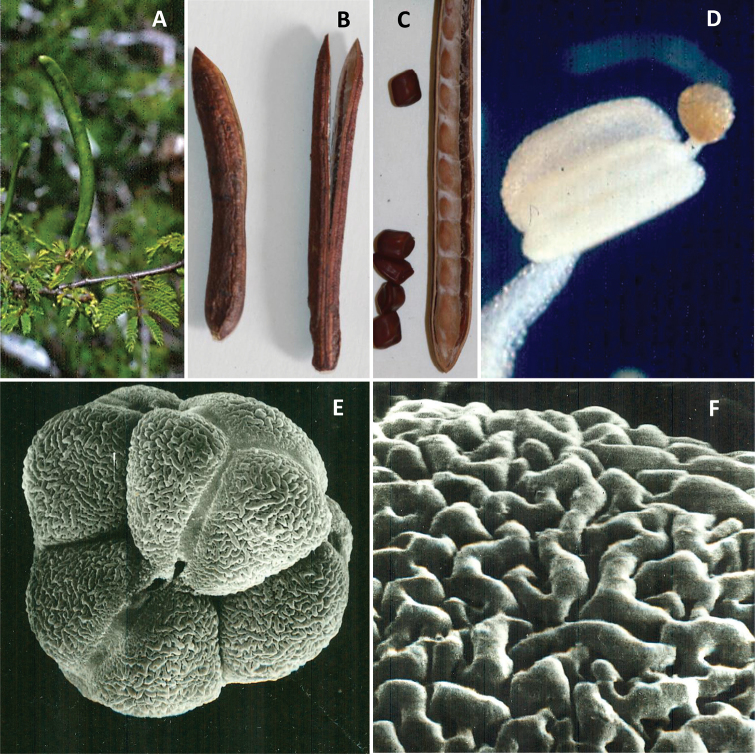
Morphology of *Mezcalabalsensis***A** unripe fruits held erect above branchlets **B, C** ripe fruits, tardily dehiscent from the apex, terete or sub-cylindrical in cross-section with thickened valves, and rhombic, four-angled seeds **D** claviform anther gland **E** tetrahedral tetrad of tricolporate pollen grains **F** exine of pollen showing striate ornamentation. Photos **A–C** José Luis Contreras Jiménez **D** Alejandro Martínez Mena, Facultad de Ciencias, Universidad Nacional Autónoma de México **E, F** Egon Köhler, Humboldt University, Berlin.

In her monograph of *Desmanthus*, Luckow (1993) pointed out that *D.balsensis* is morphologically unique within the genus. First, the fruits of *D.balsensis* are unusual within *Desmanthus*, being terete or sub-cylindrical in cross-section with thickened valves which are woody when ripe, held erect above the foliage (Fig. 2A–C), and tardily dehiscent along both sutures, the valves recurving from the apex as they open (Fig. 2B) and remaining attached at the base at least briefly after dehiscence. They are quite distinct from the dorsi-ventrally flattened fruits with chartaceous or coriaceous valves and passive dehiscence along one or both sutures which occur in the remaining species of *Desmanthus*. Second, the anthers of *D.balsensis* are capped by caducous terminal stipitate claviform glands (Fig. 2D; Luckow 1993: fig. 2F), which are lacking in the remainder of species in the genus and also absent in the genus *Kanaloa* (Lorence and Wood 1994). Third, the pollen of *D.balsensis* is also unique within the genus, being arranged in tetrahedral tetrads (Fig. 2E; Luckow 1993: fig. 3A), while the remaining species of *Desmanthus* and *Kanaloa* have eumonads. This suite of morphological character state differences resulted in placement of *D.balsensis* as sister to the rest of *Desmanthus* in cladistic analyses of morphological data (Luckow 1993: fig. 15), a set of analyses lacking the genus *Kanaloa* which had not been described at that time. These morphological differences, alongside the molecular evidence of non-monophyly, further support segregation of *D.balsensis* as a distinct genus.

An alternative generic delimitation to ensure generic monophyly would be to transfer *Kanaloa* to *Desmanthus*. However, *Kanaloa* itself has unique morphological features including tergeminately bipinnate leaves, a leaf formula not seen elsewhere in any genera of the informal Leucaena group (although not uncommon elsewhere in mimosoids); absence of an involucel of floral bracts subtending the capitula; a very broad funnelform anvil-shaped, flanged stigma on a style held below the anthers (Anna Palomino, pers. comm.), small, coriaceous, ovate or elliptic, monospermous fruits and unusual large cordiform seeds (Lorence and Wood 1994), a set of characters that clearly distinguish it from both *Desmanthus* and *Mezcala*, although the fruits of the unusual Baja California endemic *D.oligospermus* Brandegee are also monospermous and somewhat reminiscent of *Kanaloa* pods (Luckow 1993). Furthermore, this alternative generic delimitation would not solve, but rather would accentuate the morphological heterogeneity within the genus *Desmanthus*, much of which is attributable to inclusion of *D.balsensis*. In addition, subsuming *Kanaloa* into *Desmanthus* would have the undesirable impact of detracting attention from the plight of *Kanaloa* and from the ongoing conservation battle to save this monospecific Hawaiian genus. When it was first described, *Kanaloa* was known from just a handful of individuals on a sea stack off the coast of the island of Kaho’olawe, the smallest of the main islands of the Hawaiian archipelago (Lorence and Wood 1994), and it is now thought to be extinct in the wild and is the focus of ongoing *ex-situ* conservation efforts at the Olinda Rare Plant Propagation Facility, Maui.

*Mezcala* is placed in the informal Leucaena group, a subclade now composed of five genera: *Leucaena* Benth., *Schleinitzia* Warb. ex Guinet, *Mezcala*, *Kanaloa* and *Desmanthus* (Fig. 1, Table 1; Ringelberg et al. 2022), placed within the wider Dichrostachys clade sensu Koenen et al. (2020). This wider Dichrostachys clade includes many taxa with heteromorphic inflorescences comprised of variable proportions of neuter (non-functional staminate), male and hermaphrodite flowers, sometimes with showy staminodia at the base of the inflorescences which are characteristic of the clade even though not universal within it (Koenen et al. 2020). This lability in flower types within an inflorescence is mirrored in *Mezcala* and *Kanaloa*. While inflorescences of both these genera lack showy basal staminodes, inflorescences of *Mezcala* frequently include a proportion of sterile basal flowers and have highly variable ratios of sterile, male and hermaphrodite flowers, including some inflorescences with entirely sterile or male flowers (Contreras 1986). The same apparently applies to *Kanaloa*, given that, when the genus was first described, only male flowers were found (Lorence and Wood 1994). This variation is also apparent in *Desmanthus* s.s., but here a number of species have long-exserted, flattened, fused and showy staminodes at the base of the inflorescence (Luckow 1993), while *Leucaena* and *Schleinitzia* apparently lack sterile flowers. Similar evolutionary lability is apparent in other morphological characters across the genera of the Leucaena subclade, and notably pollen and anther glands. As indicated above, the occurrence of pollen in tetrahedral tetrads (Fig. 2E) and the presence of anther glands (Fig. 2D) separate *Mezcala* from *Kanaloa* and *Desmanthus* s.s. which have eumonad pollen and lack anther glands. In fact, the pollen arranged in tetrahedral tetrads with striate exine ornamentation (Fig. 2E, F), and the stipitate claviform anther glands of *Mezcala* and *Schleinitzia* species are nearly identical (Nevling and Niezgoda 1978; Luckow 1993), reflecting the sister group relationship of *Schleinitzia* to the *Mezcala* + *Kanaloa* + *Desmanthus* s.s. clade (Fig. 1; Ringelberg et al. 2022). This close similarity of *Mezcala* and *Schleinitzia* may also be related to the likely allopolyploid origin of the genus *Schleinitzia* potentially involving the ancestor of *Mezcala* as one of the parents – see below (Ringelberg et al. unpubl. data), which is also reflected in the sister group relationship between these two genera in the plastome phylogeny of Ringelberg et al. (2022). Pollen also varies within the genus *Leucaena* which includes species with tricolporate eumonads as well as others with pollen in polyads (Hughes 1997), including distinctive acalymmate polyads made up of porate monad units that are quite different from the tetrahedral tetrads of *Schleinitzia* and *Mezcala*. While most species of *Leucaena* lack anther glands, a few have rounded or ‘hooded’ apiculae that have been equated as homologous with stipitate anther glands (Hughes 1997). Thus, these five genera display a mosaic of character state combinations that reflects extensive morphological homoplasy across this clade, as well as apparently complex and poorly understood variation in the reproductive biology of these species involving presence or absence of anther glands, presence or absence of pollen aggregated into polyads, and highly variable ratios of sterile, male and hermaphrodite flowers within an inflorescence (Table 1).

**Table 1.** Morphological differences among genera of the Leucaena subclade.

	*Mezcala*	*Desmanthus*	*Kanaloa*	*Schleinitzia*	*Leucaena*
Anthers	glabrous; stipitate, claviform, caducous anther glands	glabrous; anther glands absent	glabrous; anther glands absent	glabrous; stipitate, claviform, caducous anther glands	often hairy; anther glands mainly absent, some spp. with small pointed or hooded apiculae
Stigma	porate	porate	broad funnelform, anvil-shaped	porate	porate
Inflorescence	variable proportions of sterile, male and hermaphrodite flowers; showy staminodes absent	variable proportions of sterile, male and hermaphrodite flowers; most spp. with exserted flattened showy staminodes	variable proportions of sterile, male and hermaphrodite flowers; showy staminodes absent	sterile flowers and showy staminodes absent	sterile flowers and showy staminodes absent
Pollen	tetrahedral tetrads	monads	monads	tetrahedral tetrads	mainly monads, three spp. with polyads of two types
Fruits	terete / sub-cylindrical, linear, valves woody, apically dehiscent	plano-compressed, linear, valves chartaceous, inertly dehiscent along both sutures	plano-compressed, small, ovate / elliptic, valves chartaceous, inertly dehiscent, monospermous	plano-compressed, linear-oblong valves coriaceous, winged, functionally indehiscent	plano-compressed, linear, valves chartaceous or coriaceous, inertly dehiscent along one or both sutures
Polyploidy	not polyploid	not polyploid	not polyploid	likely paleo-allopolyploid	paleopolyploid & five neotetraploid spp.

Reticulation may also have contributed to the morphological homoplasy across the genera of the Leucaena subclade, with independent whole genome duplications subtending two of the genera, *Schleinitzia* and *Leucaena*, which, in the case of *Schleinitzia*, is suggested to have involved an allopolyploid event most likely involving parental lineages from the *Mezcala* + *Kanaloa* + *Desmanthus* clade, one of which was likely the ancestor of *Mezcala* (Ringelberg et al. unpubl. data).

Mexico has been an important centre of legume diversity potentially throughout the Cenozoic (Centeno-González et al. 2021), and apparently had an especially rich Oligocene fossil legume flora that included many elements assigned to subfamily Caesalpinioideae and the mimosoid clade (Calvillo-Canadell and Cevallos-Ferriz 2005; Magallón-Puebla and Cevallos-Ferriz 1994), and Mexico remains an extremely important global centre of legume diversity today (Sousa and Delgado 1993). The segregation of *Mezcala* as a distinct genus adds to the tally of legume genera endemic to Mexico. This includes three other genera in subfamily Caesalpinioideae – *Heteroflorum* M. Sousa, *Conzattia* Rose and *Calliandropsis* H.M. Hern. and P. Guinet – which are also monospecific, and which also grow in similar seasonally dry tropical forest and scrubland habitats to *Mezcala* in south-central Mexico. Age estimates for the divergence times of these three monospecific genera and for *Mezcala* from their sister groups are strikingly congruent, all of them falling in the mid- to late-Miocene, 11–16 Myr (Ringelberg et al. in prep). This shows that these depauperon Mexican endemic dry habitat Caesalpinioid legume lineages are palaeoendemics and suggests they may be best viewed as potential relics of a formerly richer Oligocene / early Miocene Mexican seasonally dry tropical legume flora. All this further emphasizes the conservation importance of *Mezcala* and of the rich diversity of distinctive and deeply divergent legume lineages endemic to Mexico more generally (Sousa and Delgado 1993).

### ﻿Key to the genera of the Leucaena subclade (see also Table 1)

**Table:** 

1	Stipitate, terminal, claviform (orbicular on a filiform stalk) anther glands present	**2**
–	Anther glands lacking, or reduced to small protrusions (apiculae) on the apex of the anthers	**3**
2	Fruits dorsi-ventrally flattened, functionally indehiscent, with slightly winged valves that split along both sutures but do not separate over the seed chambers; widespread across the western Pacific Basin (New Guinea, Melanesia, Micronesia, Polynesia)	***Schleinitzia***
–	Fruits sub-cylindrical, tardily dehiscent along both sutures from the apex; endemic to the Balsas Depression in south-central Mexico	***Mezcala***
3	Leaves tergeminately bipinnate, i.e. with a single pair of pinnae, each with three leaflets; endemic to Hawaii	***Kanaloa***
–	Leaves almost always with > 1 pair of pinnae, each pinna with ≥ 2 pairs of leaflets and generally > 5 pairs, and often many more, never tergeminate; widespread across the Americas	**4**
4	Stipules simple, ovate or lanceolate, the mid vein visible with variably sized membranous wings on either side, sterile flowers generally lacking and flowers never with long-exserted staminodia, anthers often hairy	***Leucaena***
–	Stipules setiform with auriculate, erose, membranous, striately veined wings at the base, in some species the auricles developed into a tooth that curls under the petiole, capitula typically with a proportion of sterile flowers basally and these often with long-exserted, flattened, fused and sometimes showy staminodia, anthers glabrous	***Desmanthus***

## ﻿Taxonomy

### ﻿ Mezcala

Taxon classificationPlantaeFabalesLeguminosae

C.E. Hughes & J.L. Contr. gen. nov.

729BDA12-B231-5CE2-9B7A-8F9C01F943B2

urn:lsid:ipni.org:names:77303768-1

#### Diagnosis.

*Mezcala* is distinguished from *Desmanthus* s.s. and *Kanaloa* by the presence of a claviform anther gland with an orbicular head on a filiform stalk on the apex of the anthers, this best seen in bud and often caducous after anthesis, versus absence of anther glands; by the aggregation of pollen into tetrahedral tetrads as opposed to pollen shed as eumonads; and by its sub-cyclindrical, lignified fruits that are held erect above the shoots and which are tardily dehiscent along both sutures from the apex as opposed to the dorsi-ventrally flattened pods with chartaceous or coriaceous valves and passive dehiscence found in species of *Desmanthus* s.s and *Kanaloa*.

#### Type.

*Mezcalabalsensis* (J.L. Contr.) C.E. Hughes & J.L. Contr. = *Desmanthusbalsensis* J.L. Contr.

#### Description.

(modified from Luckow 1993: 59–60). Small multi-stemmed erect treelet or large shrub 1–3 m tall. Young shoots angled, woody, glabrous or with amorphous red glandular protrusions, reddish-brown when very young, soon exfoliating a waxy white cuticle; older stems terete, reddish-brown to grey, wrinkled, glabrous with conspicuous lenticels, branches geniculate; trunks with checkered grey bark. Stipules persistent, 1.5–3 mm long, setiform with striate, membranous wings, glabrous, red or green, the fused bases clothing short shoots on the older branches from which new leaves or side shoots arise. Leaves 2.5–4.5 cm long, petiole 5–9 mm long, rachis 11–18 mm long, red granular tissue scattered along the axes and concentrated at the junctions of the leaflets with the pinna, and pinnae with the rachis; pinnae 2–4 (–5) pairs, 9–20 mm long, the lowest pair bearing a stipitate nectary 0.4–0.7 mm in diameter on a 0.5–1 mm-long stipe, the tip orbicular, crateriform and flared; leaflets 8–14 pairs per pinna, inserted several millimeters above the base of the pinna, shortly petiolate, 2.5–3.5 × 0.8–1.2 mm, oblong, oblique to square basally, the apex acute, glabrous, finely ciliate along the margins, venation obscure except the nearly central midvein. Capitula 1–2 per leaf axil, borne on peduncles 1–3 cm long. Bracts subtending each flower 1–2.5 × 0.25–0.5 mm, deltate setiform, pale reddish or purple when dry, membranous with a single opaque midvein, peltate and short pedicellate at the centre of the capitulum, sessile at the base, persistent. Flower buds obovate, apically rounded. Capitula 0.5–1 cm long, containing 30–50 sterile, functionally male and hermaphrodite flowers, sterile or male flowers rarely absent, proportions of each flower type variable. Sterile flowers 0–5; calyx 1–1.75 × 0.5–1 mm, obconic, minutely 5-lobed; petals 2–2.5 × 0.2–0.4 mm, lanceolate, white or pale green; staminodia 10, 2.5–5 mm long, the same widths as the filaments of functional stamens, white. Male flowers 12–30, borne above the sterile flowers but with a perianth and androecium like that of the hermaphrodite flowers. Hermaphrodite flowers 5–25; calyx 1.4–2.7 mm long, obconic, the tube 1.3–2 mm long, 0.8–1.2 mm in diameter, rimmed with 5 free acute lobes 0.3–0.5 mm long; petals 2–3.5 × 0.3–0.5 mm, oblanceolate, pale green with white margins, glabrous; stamens 10; 3.5–5.5 mm long, anther apically with a minute orbicular gland borne on a filiform stalk, caducous; ovary 1–1.5 mm long, linear, glabrous, style 3.5–6 mm long, always more than three times the length of the ovary, exserted beyond the stamens. Fruiting peduncles 1–3 cm long, bearing 1 (–4) pods held erect above shoots and tardily dehiscent from the apex along both sutures, also splitting irregularly and transversely along valves, 3.2–5.5 (–10) × 3.3–5 × 0.25–5 mm, linear-oblong, straight to slightly arcuate, apex acute, valves initially fleshy, glabrous, bright emerald-green when unripe, becoming woody or sub-woody and turning dark brown when ripe. Seeds 5–13 per pod, 4.4–6 mm × 2.5–3.5 mm, longitudinally inserted, square to rhomboidal, 4-angled, deep reddish-brown; pleurogram 0.5–1 mm wide, 0.7–1.5 mm deep, deeply U-shaped, often asymmetric with unequal arms.

#### Geographic distribution.

**Figure 3.**
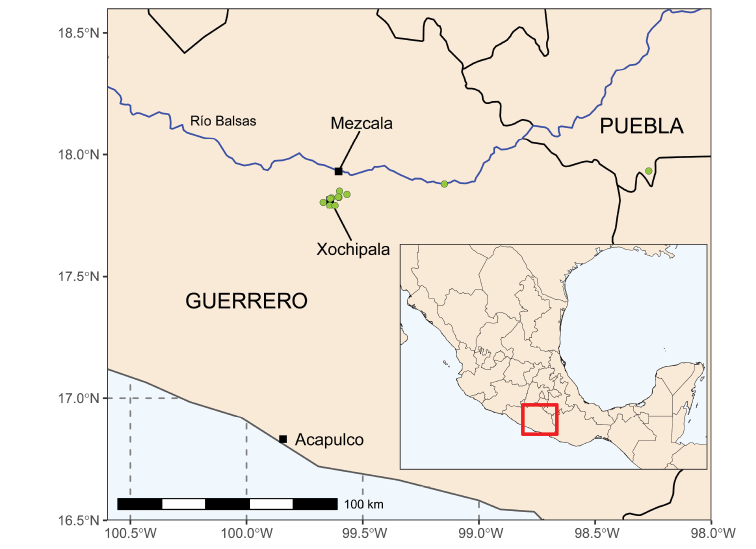
Distribution of *Mezcalabalsensis* in the central Balsas Depression in Guerrero, Mexico. Map based on 15 quality-controlled species occurrence records from GBIF (www.gbif.org), SEINet (www.swbiodiversity.org/seinet), and Contreras (1986), created using R packages ggplot2 (Wickham 2016), sf (Pebesma 2018), and rnaturalearth (South 2017), with data layers depicting Río Balsas and borders of Mexican states downloaded from the North American Environmental Atlas (www.cec.org/north-american-environmental-atlas).

*Mezcala* is a narrowly restricted endemic genus, known from just a handful of localities in the central Balsas Depression in Guerrero, Mexico (Fig. 3). A large majority of the collections are from karst limestone ridges above the gorge of the Río Xochipala, a few km from the village of Xochipala in the Municipio Eduardo Neri, with two outlying localities to the east, close to Tlalcozotitlán, in Municipio Copalillo, and south-east of Olomatlán, in Municipio Tecomatlán, in the extreme south-east of the State of Puebla. Given that *Mezcala* is undoubtedly globally rare, with an extremely restricted range, and is only known from a handful of populations, it is clear that the conservation status of the genus, although not formally assigned an IUCN threat category here, is likely to be vulnerable or potentially endangered.

#### Habitat.

Locally common, or in places close to Xochipala even abundant, in typical succulent-rich, grass-poor, seasonally dry deciduous tropical forest (SDTF) and dry scrubland with *Bursera* Jacq. ex L. (Burseraceae), *Bourreria* P. Browne (Boraginaceae), *Neobuxbaumiamezcalaensis* (Bravo) Backeb. (Cactaceae), and *Bauhiniaandrieuxii* Hemsl., *Conzattiamultiflora* (B.L. Rob.) Standl., *Haematoxylumbrasiletto* H. Karst., *Lysilomatergeminum* Benth. and species of *Mimosa* L. (all Leguminosae), on dry karst limestone with shallow freely drained soils.

#### Etymology.

*Mezcala* is named with reference to the indigenous Mezcala culture, which like the genus *Mezcala* itself, is little-known, elusive, distinctive and narrowly endemic to central Guerrero, and which blossomed in this area 700–200 BC. Vestiges of the Mezcala culture are found today along the Río Balsas and its tributaries (Reyna Robles 2020), including an important archaeological site at Xochipala, the type locality of *M.balsensis*. This is the second mimosoid legume genus named after an indigenous Mexican cultural group following the earlier example of Hernández (1986) who coined the generic name *Zapoteca* H.M. Hern. Adding a second name of similar derivation recognizes the diversity and importance of, and threats to, both endemic legumes and indigenous cultures in Mexico.

### ﻿ Mezcala balsensis

Taxon classificationPlantaeFabalesLeguminosae

(J.L. Contr.) C.E. Hughes & J.L. Contr. comb. nov.

3C139BB4-CEE8-50ED-826C-60B27B4C2DCA

urn:lsid:ipni.org:names:77303769-1

#### Basionym.

*Desmanthusbalsensis* J.L. Contr., Phytologia 60 (2): 89. (1986).

#### Type.

Mexico, Guerrero, Mpio. Zumpango del Río, 4 km ENE of Xochipala, 7 Nov. 1985, *Contreras 1737* (holotype: FCME!; isotypes: MEXU – 2 sheets!, MO, TEX!).

## Supplementary Material

XML Treatment for Mezcala

XML Treatment for Mezcala balsensis
